# Common Risk Factors for CD4 Cell Count and Hemoglobin Level among Female Adult HIV-Positive Patients: A Retrospective Longitudinal Study

**DOI:** 10.1155/2024/8461788

**Published:** 2024-09-05

**Authors:** Nurye Seid Muhie

**Affiliations:** Department of Statistics Mekdela Amba University, Tulu Awulia, Ethiopia

## Abstract

**Background:**

HIV is one of the most significant worldwide health concerns of the twenty-first century and a serious threat to human society. Hemoglobin level and CD4 cell count are two of the most important biomarkers of HIV progression and patient survival. The objective of this study was to identify common risk factors associated with CD4 cell count and hemoglobin level among adult female HIV-positive patients treated with ART at the University of Gondar Comprehensive Specialized Hospital, Ethiopia.

**Methods:**

The source of data in this study was secondary data conducted in the University of Gondar Comprehensive Specialized Hospital from September 2015 to March 2022 . Data exploration in this study was normal histogram plot, box plot, and Q-Q plot considered to gain some visions of the data related to CD4 cell count and hemoglobin level. A Bayesian joint model was used in this longitudinal data set to get a wide range of information about adult female HIV-patients.

**Results:**

The mean with a standard deviation of hematocrit (%), red blood cell (10^6^/*μl*), lymphocyte (%), and weight (kg) of female patients were 37.2 (3.8), 4.0 (1.6), 43.6 (11.8), and 44.9 (9.4), respectively. In this study, the random intercept model for CD4 cell count and the random intercept and slope model for hemoglobin level were considered as the best selected model. Visit time, hematocrit, weight, RBC, lymphocyte count, educational status, marital status, disclosure, and substance use were common risk factors for CD4 cell count and hemoglobin level.

**Conclusion:**

This study concluded that, the risk factors visit time, weight, secondary educators, tertiary educators, married patients, patients who disclosed their HIV status to family members were associated with high CD4 cell count and hemoglobin level. While, hematocrit, RBC, lymphocyte count, separated marital status, widowed marital status, and substance-addicted patients were associated with low CD4 cell count and hemoglobin level. The author recommended that FMOH or other health professionals, program planners, decision makers, project implementers, government, and nongovernmental organizations should be given special attention for adult female patinets to minimize the risk of HIV progression and improve their health status. The author also recommended that health staff should conduct health-related studies for patients to examine continuous checkups. Health professionals also should give more attention to types of ART medication to reduce the progression of disease when the patients come back again into the hospital. Finally, adult female HIV-positive patients should be given special attention based on these important factors to improve their CD4 cell count, hemoglobin level, and better health quality.

## 1. Background

HIV is one of the most significant worldwide health concerns of the twenty-first century and a serious threat to human society. The virus continues to be a serious global public health concern, having taken 40.4 million lives so far and continuing to spread throughout all nations. By the end of 2022, there were an anticipated 39.0 million HIV-positive individuals worldwide, with 25.6 million of them individuals living in Africa. In 2022, 1.3 million new cases of HIV infection and 630,000 deaths from HIV-related causes were reported [1].

Globally, the proportion of people living with HIV is decreasing, and many affluent countries are managing their HIV epidemics. However, several developing countries, including Ethiopia, are increasing the frequency of HIV infection [2].

Antiretroviral therapy (ART) has been used to help HIV-infected patients live healthy with better survival [1]. Then, hemoglobin and CD4 cell count are two of the most important biomarkers of HIV infection prognosis and patient survival. However, the risk of death for AIDS patients rises when the quantity of hemoglobin level and CD4 cell count declines [3, 4]. Even after beginning antiretroviral medication, HIV-infected patients are at risk of death because of decreased hemoglobin levels and CD4 cell counts [5, 6].

HIV significantly impairs the immune system below 100 cells/mm^3^, which is a sign of immunological failure in individuals whose CD4 cell count is less than 500 cells/mm^3^ [7]. A hemoglobin content below 13 g/dL for men and 12 g/dL for women may indicate an infection-related illness [8]. On the other hand, adult HIV-positive patients have lower hemoglobin levels [9] and CD4 cell counts than the expected healthy adults, which indicates a higher risk of HIV progression [10].

The majority of earlier studies were done on CD4 cell count and hemoglobin level separately. Some of the studies done separately indicated that risk factors associated with female adult patients' CD4 cell count were age, weight, baseline CD4 cell count, cell phone ownership, visit time, marital status, residence, and level of disclosure of the disease to family members [11], household income, WHO clinical stage, ART adherence [12], and opportunistic infections [13].

Another study also indicated that the risk factors for female adult patients' hemoglobin level were sex, age, hepatitis C virus coinfection, higher viral load, 1c ART treatment, illicit drug, BMI <18.5 kg/m^2^, BMI between 25 and <30 kg/m^2^, BMI ≥30 kg/m^2^, smoker, alcohol addicted [14], weight, CD4 cells <350/mm^3^ [15], red blood cell count, platelet cell count, lymphocyte count [9], tuberculosis co-infection, advanced WHO stage [16], other opportunistic infections, poor adherence to ART, rural residence, and eating nondiversified foods [17].

Therefore, to the extent that various studies happened as some indicated above for analyzing risk factors for CD4 cell count, and hemoglobin level separately, there is a scarcity of studies now conducted for CD4 cell count and hemoglobin level jointly among adult female HIV-positive patients. Then, to fill the gap between any previous related studies, the objective of this study was to identify common risk factors associated with CD4 cell count and hemoglobin level among adult female HIV-positive patients treated with ART at the University of Gondar Comprehensive Specialized Hospital (UGCSH), Ethiopia. The findings from this study will need to be a source for other researchers, the body of knowledge that informs HIV program planners, decision makers, and project implementers by providing common risk factors of CD4 cell count and hemoglobin level among adult female HIV-infected patients.

## 2. Material and Methods

### 2.1. Study Area

This study was conducted at the University of Gondar Comprehensive Specialized Hospital (UGCSH).

### 2.2. Study Design

A retrospective cohort follow-up study was carried out to retrieve relevant information from the medical records of adult female HIV-positive patients.

### 2.3. Study Population

The study population for this study was all adult female HIV-positive patients under reproductive age groups.

### 2.4. Study Period

The study period was female patients who started ART treatment from September 2015 to March 2022.

### 2.5. Source of Data

The source of data in this study was secondary data obtained from patients' charts.

### 2.6. Inclusion Criteria

This study included all adult female HIV-positive patients under reproductive age groups who had at least a minimum of two visits (one year) for repeated measurements (CD4 cell count and hemoglobin level), patients whose age groups between 15 and 45 years, and patients who had started ART treatment within the treatment follow-up study period.

### 2.7. Exclusion Criteria

This study excluded patients under reproductive age groups who had only one visit for repeated measurements (CD4 cell count and hemoglobin level), patients whose age groups were outside 15 and 45 years, and patients who had started ART treatment without the study period.

### 2.8. Data Collection Procedure

The data collection procedures in this retrospective cohort study were based on the patient's chart and electronic database system (smart care). Using the medical registration number (MRN) of patients from database system, patient charts can be selected, and from the review of patient charts, the necessary information was retrieved by two trained ART data clerks who were trained in ART data management.

### 2.9. Data Collection Quality

One day of intensive training was given to ensure the quality of collected data. Before the actual data were collected, the adequacy of the checklist was evaluated, and ambiguous questions were modified. The necessary amendments are made to the final data extraction format for completeness and consistency, and the full formats are checked by ART data management.

### 2.10. Variables Included in the Study

#### 2.10.1. Response Variable

The response variable in this study was CD4 cell count in cells/mm^3^ and hemoglobin level in grams per deciliter (g/dl).

#### 2.10.2. Independent Variable

The independent variable in this study was commonly affecting CD4 cell count and hemoglobin level. Thus, sociodemographic, behavioral, and clinical variables were visit time, viral load count, white blood cell (WBC), red blood cell (RBC), platelet cell count, hematocrit, monocyte count, lymphocyte count, weight, BMI, age, functional status, residence, religion, marital status, educational status, disclosure status, treatment adherence, TB screen, opportunistic infections (OIs), WHO clinical stage, other comorbid condition (OCC), and substance use.

### 2.11. Method of Data Analysis

The method of data analysis was done by R software version based on a 5% level of significance for statistical decisions.

### 2.12. Exploratory Data Analysis for Normality Assumption

Data exploration highlights the nature of the data. In this study, normal histogram plot, box plot, and Q-Q plot were considered to gain some visions of the data related to CD4 cell count and hemoglobin level.

### 2.13. Statistical Model

In this study, a Bayesian linear mixed model was used jointly for repeated measure CD4 cell count and hemoglobin level.

### 2.14. Variable Selection

Variable selection is used to find a subset of the accessible contributions that precisely predict the outcome. Purposeful variable selection is appropriate for covariates and can be used by deciding on each step of the modeling process at a 25% level of significance. In this study, a purposeful variable selection method was used to select appropriate explanatory variables.

### 2.15. Model Selection Criteria

To select the better model which appropriate to the given data, it is necessary to compare different models by using different techniques and methods. In this study, the deviance information criteria (DIC) are the most commonly used method of model selection criteria. Therefore, the model with the smallest value of DIC is the appropriate model.

## 3. Results

### 3.1. Clinical Characteristics of Female Adult HIV-Infected Patients

Out of 201 study participants incorporated in this study, those without TB were 163 (81.1%), of which 25 (15.3%) died from HIV. Less than one-third of female participants (28.9%) had other comorbid conditions (OCC), of which 15.5% had mortality from the disease. Likewise, less than one-fourth of female patients (25.9%) were affected by opportunistic infections (OIs) other than TB, of which 10 (19.2%) had mortality from the disease. Considering the WHO clinical stage of female participants, 111 (55.2%), 31 (15.4%), 33 (16.4%), and 26 (12.9%) were Stage I, Stage II, Stage III, and Stage IV, respectively. Around 26.4% of the study participants had fair treatment adherence status, of which 47.2% had the outcome of mortality. The minimum values of viral load count in copies/mL, hematocrit in %, WBC in 10^3^/*μ*l, RBC in 10^6^/*μ*l, platelet cell count in 10^3^/*μ*l, lymphocyte count in %, and monocyte count in % were 11, 27.9%, 2.6, 2.0, 23, 21.0, and 2.6, respectively. Similarly, the maximum value of viral load count in copies/mL, hematocrit in %, WBC in 10^3^/*μ*l, RBC in 10^6^/*μ*l, platelet cell count in 10^3^/*μ*l, lymphocyte count in %, and monocyte count in % were 5623, 54.0, 11.0, 9.0, 583, 69.1, and 13.4, respectively (Table 1).

### 3.2. Sociodemographic Characteristics of Female Adult HIV-Infected Patients

More than half of the female patients (60.2%) in this study can be considered as the age group between 25 and 34 years, of which 18.2% died during the follow-up period. Considering the residence of female patients, almost more than half of the patients (52.2%) were rural residents, of which 20.0% lead to death from disease. Furthermore, in terms of educational status, 19.9%, 34.3%, 31.3%, and 14.4% were noneducators, primary, secondary, and tertiary patients, respectively. Similarly, 87.6% disclosed the disease to family members, and out of these participants, 17.6% died from the disease. Regarding substance use, more than half (70.6%) were classified as nonsubstance users, of which 8.5% of mortality were from HIV. Considering the marital status of female patients, almost more than half of the female patients (50.2%) were married, and 25.7% died from the disease. The majority of the patients (91%) in this study can be considered orthodox religious followers. The minimum, maximum, mean, and standard deviation for body weight were 33.4, 84, 44.9, and 9.4 kg, respectively. Similarly, BMI 11.4, 34.5, 18.2, and 4.2 kg/m^2^ were considered as minimum, maximum, mean, and standard deviation, respectively (Table 2).

### 3.3. Exploratory Data Analysis for Normality Assumption

**Figure 1:**
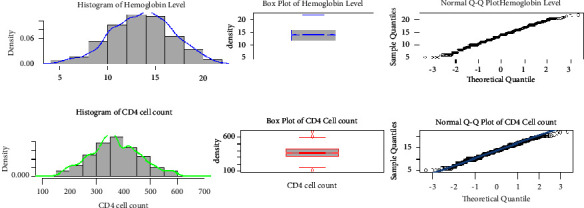
Histogram, box plot, and Q-Q plot for CD4 cell count and hemoglobin.

The normality assumption for repeated measure CD4 cell count and hemoglobin was satisfied by constructing different plots like histograms, box plots, and normal Q-Q plots (Figure 1). Hence, the data set can be performed without any transformation techniques.

### 3.4. Random Effect Model Selection

From Table 3, the full model was fitted with considered all covariates, and the null model was the model fitted without covariates. The deviance information criteria (DIC) can be used to select the most appropriate random effect model. Hence, the full model (model III) with random intercept for CD4 cell count and random intercept and slope for hemoglobin level was a better-fitted model (Table 3).

### 3.5. Univariable Variable Selection

At a 25% level of significance by using the purposeful variable selection, the covariate age, educational status, adherence, hematocrit, weight, BMI, WBC, RBC, platelet cell count, lymphocyte, monocyte, functional status, OIs, OCC, residence, religion, marital status, substance use, and disclosure are statistically significant risk factors for CD4 cell count. However, the remaining covariates were not considered as a significant risk factors for CD4 cell count.

Likewise, the covariate educational status, adherence, hematocrit, weight, BMI, WBC, RBC, lymphocyte, functional status, OCC, WHO clinical stage, religion, marital status, substance use, and disclosure were considered statistically significant risk factors for hemoglobin level. Conversely, the remaining covariates had a statistically not significant effect on hemoglobin level.

Bivariate analysis showed that the covariate visit time in month, hematocrit, weight, WBC, RBC, platelet cell count, lymphocyte count, monocyte count, age in year, treatment adherence, OIs, religion, marital status, educational status, disclosure, and substance use were significantly affected repeated measure CD4 cell count of female adult HIV-positive patients (Table 4). Similarly, visit time in month, hematocrit, weight, BMI, RBC, lymphocyte count, WHO clinical stage, functional status, OCC, marital status, educational status, disclosure, and substance use were significantly affected repeated measuring hemoglobin levels of female adult HIV-positive patients (Table 5).

The result of two longitudinal joint models indicated visit time, hematocrit, weight, RBC, lymphocyte count, educational status, marital status, disclosure, and substance uses were considered to be common significant risk factors for repeated measure CD4 cell count and hemoglobin level (Tables 4 and 5).

## 4. Discussion

As far as we are aware, this is the first study on the common risk factors of female adult HIV-positive patients' hemoglobin level and CD4 cell count at the University of Gondar Compressive Specialized Hospital. The purpose of this study was to identify the common factors that influenced repeated measures of hemoglobin level and CD4 cell count. Then, this result demonstrated that the most significant common risk factors of CD4 cell count and hemoglobin level are visit time, hematocrit, weight, RBC, lymphocyte, educational status, marital status, disclosure, and substance use.

Female tertiary educators (*β* = 1.6097, 95% CI (1.0982–2.1211)) had incremented in their average CD4 cell count by 1.6 cells/mm^3^ than noneducators. The finding of this study is consistent with a previous study [18]. Likewise, secondary and tertiary female educators (*β* = 0.2690, 95% CI (0.2436–0.2945)) and (*β* = 0.5436, 95% CI (0.5148–0.5724)) had incremented by 0.3 g/dl and 0.5 g/dl in their average hemoglobin level than noneducator patients, respectively. This study contradicts the previous study done in Ethiopia [19]. The result of this study shows that higher educational levels of female patients show increment variations of CD4 cell count and hemoglobin level due to a better understanding of HIV progression, and ART treatment follow-up leads to good survival of life. This study demonstrates the advantages of education for females with HIV are improved access to health services, decreased social and economic vulnerability that exposes women to risky activities, and an increased chance of joining community groups that promote AIDS prevention. The finding of this study is in line with a previous study [20].

Married and separated female patients (*β* = 1.5479, 95% CI (1.4903–1.6055)) and (*β* = −3.1813, 95% CI −(3.3657−2.9969)) had incremented by 1.5 cells/mm^3^ and decremented by 3.2 cells/mm^3^, respectively, in their average CD4 cell count than single patients. Likewise, married and widowed (*β* = 0.4695, CI (0.4454–0.4936)) and (*β* = −0.2412, 95% CI −(0.2819−0.2004)) had incremented by 0.5 g/dl and decremented by 0.24 g/dl, respectively, in their average hemoglobin level than single patients. This result indicates marriage female patients had better CD4 cell count and hemoglobin levels than single patients due to appropriate follow-up of ART treatment. However, separated and widowed female patients had lower CD4 cell count and hemoglobin levels than single patients due to poor follow-up status of ART treatment. Furthermore, separated and divorced patients' nonexistence of emotional support deserves the particular attention of HIV and leads to high disease progression. The finding of this study is in line with a previous study [21].

Female patients who disclosed the disease to a family member (*β* = 1.5777, 95% CI (1.0608–2.0946)) and (*β* = 0.4304, 95% CI (0.4014–0.4594)) had incremented average CD4 cell count and hemoglobin by 1.6 cells/mm^3^ and 0.4 g/dl, respectively, than patients who do not know disease status to family members. These suggested female HIV-positive patients might be associated with the health-related quality of life. Furthermore, patients have improved general health and sense increased social support from their extended family network. Moreover, family members can share the emotional load, which helps patients feel less depressed, worn out, and anxious. This idea is supported by the previous study [22].

Substance-addicted patients (*β* = −4.1265, 95% CI −(4.5928−3.6602)) and (*β* = −0.7820, 95% CI −(0.8002−0.7620)) had decremented in their average CD4 cell count and hemoglobin by 4.1 cells/mm^3^ and 0.8 g/dl, respectively, than patients who do not substance-addicted. These reflected HIV was a highly progressed and a high health problem for female substance-addicted patients. Females, who are addicted to alcohol and noninjection drugs, including crack cocaine, may increase a woman's risk of sexually transmitted HIV infection through increased engagement in high-risk sexual behaviors, such as unprotected sex and sex exchange for drugs. Due to this reason, patients with less CD4 cell count and hemoglobin result for poor health outcomes. The result of this study is consistent with the previous studies done on American women [21].

The other covariates were constant, hematocrit increased by one unit, the average hemoglobin level of female patients was decreased by 0.04 g/dl (*β* = −0.0366, 95% CI −(0.0386−0.0346)). These imply patient's low hematocrit that leads to low hemoglobin levels. This idea is contradicted by the previous study [23, 24]. Similarly, with one unit increase in hematocrit of female patients, the average CD4 cell count was decreased by 1.4 cells/mm^3^ (*β* = −1.3917, 95% CI −(2.2001−0.5864)). That is, low hematocrit patients lead to low repeated measure CD4 cell count and poor health improvement. This result was similar with the previous literature [23], but opposed with a study done at Haji Adam Malik General Hospital, Medan, Indonesia [25].

Female patients' red blood cell (RBC) count increased by one unit, the average hemoglobin level was decreased by 0.004 g/dl (*β* = −0.0040,  95% CI : −(0.0920 − 0.0764)). On the other hand, patients' low RBC results in low repeated measure hemoglobin levels, leading to poor health status. These findings are consistent with a previous study done by [26]. Similarly, with one unit increase in RBC of female patients, the average CD4 cell count was decreased by 1.9 cells/mm^3^ (*β* = −1.8926, 95% CI −(2.1427−0.6425)). That is, patients' RBC decreases, and repeated measure CD4 cell count becomes an abnormal condition and results in low survival time and easily leads to death. This result is contradicted by prior study [25] with results, and there was an insignificant but negative association between RBC and CD4 cell count.

The other covariates were constant, the weight of patients increased by one unit, the average CD4 cell count was increased by 2.1 cells/mm^3^ (*β* = 2.0841,  95% CI (0.0662 − 1.9543)). These imply patients with high weight, which lead to high CD4 cell count [27, 28]. Similarly, with one unit increase in weight of female patients, the average hemoglobin level was increased by 0.002 g/dl (*β* = 0.0022, 95% CI (0.0096–0.0182)). That is, patient's weight is high and results in a high repeated measure hemoglobin level. The findings of this study are in line with a previous study [29].

Among the lymphocyte count of patients, the average CD4 cell count was decreased by 0.8 cells/mm^3^(*β*=−0.8289,  95% CI  − (0.8538 − 0.8040)) as one unit increased lymphocyte. Likewise, with one unit increase in lymphocyte count, the average hemoglobin level was decreased by 0.03 g/dl (*β* = −0.0264, 95% CI −(0.0272−0.0256)). These indicated that female patients' lymphocyte count was small and poor repeated measure of CD4 cell count and hemoglobin level. The result of these studies was supported by the previous literature [30, 31].

## 5. Conclusions

This study concluded that, the risk factors visit time, weight, secondary educators, tertiary educators, married patients, patients who disclosed their HIV status to family members were associated with high CD4 cell count and hemoglobin level. While, hematocrit, RBC, lymphocyte count, separated marital status, widowed marital status, and substance-addicted patients were associated with low CD4 cell count and hemoglobin level. The author recommended that FMOH or other health professionals, program planners, decision makers, project implementers, government, and nongovernmental organizations should be given special attention for adult female patinets to minimize the risk of HIV progression and improve their health status. The author also recommended that health staff should conduct health-related studies for patients to examine continuous checkups. Health professionals also should give more attention to types of ART medication to reduce the progression of disease when the patients come back again into the hospital. Finally, adult female HIV-positive patients should be given special attention based on these important factors to improve their CD4 cell count, hemoglobin level, and better health quality.

## Figures and Tables

**Table 1:** Baseline clinical characteristics of patients.

Variables	Categories	Total (%)	Survival status	
			Censored (%)	Death (%)

TB screen	No	163 (81.1)	138 (84.7)	25 (15.3)
	Yes	38 (18.9)	25 (65.8)	13 (34.2)

OCC	No	143 (71.1)	114 (79.9)	29 (20.3)
	Yes	58 (28.9)	49 (84.5)	9 (15.5)

OIs	No	149 (74.1)	121 (81.2)	28 (18.8)
	Yes	52 (25.9)	42 (80.8)	10 (19.2)

WHO clinical stage	Stage I	111 (55.2)	102 (91.9)	9 (8.1)
	Stage II	31 (15.4)	25 (80.6)	6 (19.4)
	Stage III	33 (16.4)	28 (84.8)	5 (15.2)
	Stage IV	26 (12.9)	8 (30.8)	18 (69.2)

Adherence	Poor	12 (6.0)	5 (41.7)	7 (58.3)
	Fair	53 (26.4)	28 (52.8)	25 (47.2)
	Good	136 (67.6)	130 (95.6)	6 (4.4)

Continuous variables	Minimum	Maximum	Mean	Standard deviation

Viral load count	11	5623	575.88	1017.18
Hematocrit in %	27.9	54.0	37.2	3.8
WBC in 10^3^/*μ*l	2.6	11.0	6.1	1.8
RBC in 10^6^/*μ*l	2.0	9.0	4.0	1.6
Platelet in 10^3^/*μ*l	23	583	284.2	95.5
Lymphocyte in %	21.0	69.1	43.6	11.8
Monocyte in %	2.6	13.4	7.9	3.1

TB: tuberculosis, OCC: other comorbid condition, and OIs: opportunistic infections.

**Table 2:** Baseline sociodemographic and behavioral characteristics of patients.

Variables		Categories	Total (%)	Survival status	
				Censored (%)	Event (%)

Age		15–24 years	34 (16.9)	28 (82.4)	6 (17.6)
		25–34 years	121 (60.2)	99 (81.8)	22 (18.2)
		35–45 years	46 (22.9)	36 (78.3)	10 (21.7)

Residence	Urban		96 (47.8)	79 (82.3)	17 (17.7)
	Rural		105 (52.2)	84 (80.0)	21 (20.0)

Level of education		Noneducator	40 (19.9)	34 (85.0)	6 (15.0)
		Primary	69 (34.3)	57 (82.6)	12 (17.4)
		Secondary	63 (31.3)	50 (79.4)	13 (20.6)
		Tertiary	29 (14.4)	22 (57.9)	7 (24.1)

Disclosure status		No	25 (12.4)	18 (72.0)	7 (28.0)
		Yes	176 (87.6)	145 (82.4)	31 (17.6)

Substance use		No	142 (70.6)	130 (91.5)	12 (8.5)
		Yes	59 (29.4)	33 (55.9)	26 (44.1)

Marital status		Single	37 (18.4)	35 (94.6)	2 (5.4)
		Marriage	101 (50.2)	75 (74.3)	26 (25.7)
		Separated	10 (5.0)	8 (80.0)	2 (20.0)
		Widowed	16 (8.0)	13 (81.3)	3 (18.8)
		Divorced	37 (18.4)	32 (86.5)	5 (13.5)

Religion		Muslim	5 (2.5)	4 (80.0)	1 (20.0)
		Orthodox	183 (91.0)	148 (80.9)	35 (19.1)
		Other	13 (6.5)	11 (84.6)	2 (15.4)

Functional status		Working	119 (59.2)	107 (53.2)	12 (6.0)
		Ambulatory	67 (33.3)	44 (65.7)	23 (34.3)
		Bedridden	15 (7.5)	12 (80.0)	3 (20.0)

Continuous variables		Minimum	Maximum	Mean	Standard deviation

Weight in kg		33.4	84.0	44.9	9.4
BMI in kg/m^2^		11.4	34.05	18.2	4.2

**Table 3:** Random effect model selection for bivariate longitudinal response.

Model	Response variable	Random effect model		Null model	Full model
		Random intercept	Random intercept and slope	DIC	DIC
I	CD4 cell	CD4 cell	—	18252.48	18190.35
	Hgb level	Hgb	—		

II	CD4 cell	Hgb	—	18254.95	18302.23
	Hgb level	—	CD4 cell		

III	CD4 cell	CD4 cell	—	18103.44	**18055.42**
	Hgb level	—	Hgb		

IV	CD4 cell	—	CD4 cell	18268.26	18261.45
	Hgb level	—	Hgb		

CD4: a cluster of differentiation 4, Hgb: hemoglobin, and DIC: deviance information criteria. The bold value indicates better smallest DIC values.

**Table 4:** Results for bivariate joint longitudinal CD4 cell count.

Variables	Categories	Post mean	Standard error	95% CI		*p* values
				Lower	Upper	
Intercept	—	3.8666	0.3114	3.2563	4.4769	0.002^∗^

Visit time	—	1.6696	0.0042	1.9543	2.3950	0.001^∗^

Hematocrit	—	−1.3917	0.0133	−2.2001	−0.5864	0.001^∗^

Weight	—	2.0841	0.0662	1.9543	2.2138	0.040^∗^

BMI	—	1.6066	0.1557	−1.0783	7.7584	0.132

WBC	—	1.7089	0.0958	1.5211	1.8967	0.018^∗^

RBC	—	−1.8926	0.1276	−2.1427	−1.6425	0.034^∗^

Platelet	—	0.1603	0.0017	0.1570	0.1636	0.002^∗^

Lymphocyte	—	−0.8289	0.0127	−0.8538	−0.8040	0.005^∗^

Monocyte	—	−0.5057	0.0564	−0.6162	−0.3952	0.006^∗^

Age (ref = 15–24 years)	25–34	−2.1458	0.2406	−2.6174	−1.6742	0.002^∗^
	35–45	3.2541	0.2721	−3.3882	29.6259	0.110

Adherence (ref = poor)	Fair	−2.7194	0.2722	−3.2529	−2.1859	0.008^∗^
	Good	1.9788	0.2416	1.5053	2.4523	0.020^∗^

Functional status (ref = working)	Ambulatory	2.2046	0.2396	−13.638	16.8909	0.764
	Bedridden	−0.8403	0.2770	−18.127	16.9968	0.944

Residence (ref = urban)	Rural	2.8744	0.2319	−11.409	16.5124	0.706

OIs (ref = no)	Yes	3.7425	0.2391	3.2739	4.2111	0.014^∗^

OCC (ref = no)	Yes	1.9180	0.2483	−13.468	17.1560	0.812

Religion (ref = muslim)	Orthodox	2.6328	0.2773	−15.683	19.8342	0.772
	Other	3.3214	0.2985	2.7363	3.9065	0.018^∗^

Educational status (ref = noneducator)	Primary	−2.5658	0.2339	−18.179	12.0271	0.744
	Secondary	−3.2902	0.2448	−17.729	11.5047	0.658
	Tertiary	1.6097	0.2609	1.0982	2.1211	0.025^∗^

Marital status (ref = single)	Marriage	1.5479	0.0294	1.4903	1.6055	0.002^∗^
	Separated	−3.1813	0.0941	−3.3657	−2.9969	0.005^∗^
	Widowed	3.4015	0.2864	−13.902	21.6198	0.732
	Divorced	0.1881	0.2297	−14.829	14.3662	0.958

Disclosure (ref = No)	Yes	1.5777	0.2637	1.0608	2.0946	0.026^∗^

Substance use (ref = no)	Yes	−4.1265	0.2379	−4.5928	−3.6602	0.025^∗^

Sigma	—	9.0392	0.0432	8.95453	9.1239	0.001^∗^

Post mean: posterior mean for the Bayesian model and CI: credible interval. *p* values indicate probability values. ^∗^Statistical significance at a 5% level of significance.

**Table 5:** Results for bivariate joint longitudinal hemoglobin level.

Variables	Categories	Post mean	Standard error	95% CI		*p* values
				Lower	Upper	
Intercept	—	12.6076	0.1536	9.3998	15.5998	0.001^∗^

Visit time	—	0.1209	0.0004	0.1068	1.0968	0.001^∗^

Hematocrit	—	−0.0366	0.0010	−0.0386	−0.0346	0.038^∗^

Weight	—	0.0139	0.0022	0.0096	0.0182	0.034^∗^

BMI	—	0.0731	0.0059	0.0615	0.0847	0.026^∗^

WBC	—	0.1081	0.0037	−0.0370	0.2771	0.176

RBC	—	−0.0842	0.0040	−0.0920	−0.0764	0.046^∗^

Lymphocyte	—	−0.0264	0.0004	−0.0272	−0.0256	0.024^∗^

Adherence (ref = poor)	Fair	−0.2467	0.0211	−1.3552	0.8499	0.674
	Good	−0.6000	0.0249	−1.6823	0.6032	0.294

WHO clinical stage (ref = stage I)	Stage II	0.7232	0.0121	0.6995	0.7469	0.046^∗^
	Stage III	−0.4611	0.0131	−0.4868	−0.4354	0.002^∗^
	Stage IV	−0.5086	0.0149	−0.6152	−0.4794	0.026^∗^

Functional status (ref = working)	Ambulatory	0.1116	0.0183	−0.9765	1.0859	0.848
	Bedridden	−0.4734	0.0111	−0.4951	−0.4516	0.015^∗^

OCC (ref = no)	Yes	0.0574	0.0095	0.0388	0.0760	0.046^∗^

Religion (ref = muslim)	Orthodox	−0.3375	0.0550	−2.0036	1.4719	0.704
	Other	−1.6677	0.0507	−3.5870	0.2003	0.094

Educational status (ref = noneducator)	Primary	0.3409	0.0147	−0.4506	1.0588	0.368
	Secondary	0.2690	0.0130	0.2436	0.2945	0.048^∗^
	Tertiary	0.5436	0.0147	0.5148	0.5724	0.023^∗^

Marital status (ref = single)	Marriage	0.4695	0.0123	0.4454	0.4936	0.018^∗^
	Separated	−0.2609	0.0208	−1.5634	0.9885	0.670
	Widowed	−0.2412	0.0230	−0.2819	−0.2004	0.004^∗^
	Divorced	−0.4300	0.0134	−1.3052	0.3682	0.278

Disclosure (ref = no)	Yes	0.4304	0.0148	0.4014	0.4594	0.026^∗^

Substance use (ref = no)	Yes	−0.7820	0.0102	−0.8002	−0.7620	0.018^∗^

Sigma	—	2.4450	0.0022	2.3102	2.5787	0.001^∗^

Post mean: posterior mean for the Bayesian model and CI: credible interval. *p* values indicate probability values. ^∗^Statistical significance at a 5% level of significance.

## Data Availability

The data used in the current investigation are available from the corresponding author and can be attached upon request. The data accessed in the current investigation complied with relevant data protection and privacy regulations.

## Abbreviations

AIDS: Acquired immune deficiency syndrome
ART: Antiretroviral therapy
BMI: Body mass index
CD4: Cluster differentiation 4
FMOH: Federal Ministry of Health
Hgb: Hemoglobin
HIV: Human immune deficiency virus
OCC: Other comorbid condition
OIs: Opportunistic infections
TB: Tuberculosis
UGSCH: University of Gondar Comprehensive Specialized Hospital
WHO: World Health Organization.

## Additional Points

*Code Availability*. All codes organized in this study are available from the corresponding author upon reasonable request. *Transparency statement*. The lead author, Nurye Seid Muhie, affirms that this manuscript is a truthful, correct, and clear account of the study being reported; that no important aspects of the study have been absent; and that any inconsistencies from the study as planned have been explained.
